# The Influence of PTSD on Problematic Internet Use among Chinese Earthquake Survivors: The Mediating Role of Fear of Missing Out and Rumination

**DOI:** 10.31083/AP46030

**Published:** 2025-08-25

**Authors:** Chen Gong, Yezi Li

**Affiliations:** ^1^School of Journalism, Fudan University, 200437 Shanghai, China; ^2^College of Literature and Media, Wenzhou University of Technology, 325027 Wenzhou, Zhejiang, China

**Keywords:** PTSD, earthquake, fear of missing out, rumination, problematic internet use

## Abstract

**Objective:**

Problematic internet use (PIU) is a general behavioral addiction and encompasses various syndromes. Previous research found that traumatic events may potentially influence or alter the propensity for PIU. This study aimed to explore the mediating role of fear of missing out (FOMO) and rumination in the influence of post-traumatic stress disorder (PTSD) on PIU among Wenchuan earthquake survivors.

**Methods:**

In the fall of 2023, 665 valid participants’ responses were selected in this cross-sectional study. The PTSD Checklist (PCL-C), FOMO Scale, Rumination Scale (RRS), and Generalized Problematic Internet Use Scale 2 (GPIUS2) were used to measure participants’ internet usage and mental state. Description analysis and structural equation model analysis were examined by using SmartPLS.

**Results:**

PTSD positively influenced FOMO (β = 0.315, *p* < 0.001), rumination (β = 0.279, *p* = 0.001), and PIU (β = 0.213, *p* < 0.001). FOMO (β = 0.08, 95% CI (confidence interval) [0.037, 0.144], *p* = 0.005) and rumination (β = 0.093, 95% CI [0.032, 0.139], *p* = 0.002) played a mediating role in the influence of PTSD on PIU. Regarding the relationship between PTSD and PIU, direct and indirect effects comprised 45.6% and 54.4%. PTSD had a positively significant effect on PIU by mediating FOMO and rumination to form a chain mediation model (β = 0.081, 95% CI [0.010, 0.039], *p* = 0.002).

**Conclusions:**

This study investigated online usage and media psychology among survivors of the Wenchuan earthquake in China. FOMO and rumination were found to be important factors influencing the relationship between PTSD and PIU. To prevent or relieve people’s PIU, we propose that medical practitioners and local government intervene on FOMO through effective measures to decrease rumination. The individual differences and specific internet platform usage that influence these psychological variables should also be further investigated in future studies.

## Main Points

1. This is the first study to integrate and examine post-traumatic stress disorder (PTSD) and problematic internet use (PIU) exploring psychological factors and core symptoms in Wenchuan earthquake survivors.

2. This study expands previous research that examined the potential contributors and fundamental symptoms associated with the development of PIU.

3. A sample of 665 Chinese earthquake survivors were included in this study.

## 1. Introduction and Background

With the swift development of the internet in recent years, the global population of internet users has increased dramatically, consequently leading to arise in research on problematic internet use (PIU) [1]. PIU is characterized as a general behavioral addiction and encompasses various syndromes, including compulsive internet use, difficulty in regulating online time, and so on [1, 2]. Previous research found that traumatic events may disrupt the normal development of people, potentially influencing or altering the propensity for PIU [2, 3]. Traumatic events, including natural disasters, abuse, and accidents, can serve as significant external stimuli for PIU, possibly provoking various adverse psychological responses and yielding diverse psychological consequences.

Individuals might exhibit a range of adverse psychological reactions to traumatic events. Post-traumatic stress disorder (PTSD) is delayed and persistent manifestations of trauma and stressor-related disorders arising from threats or catastrophic events [4]. On May 12, 2008, Wenchuan County in Sichuan Province, China, suffered a significant earthquake measuring 8.0 in magnitude. According to Fan *et al*. [5], this earthquake caused nearly 100,000 deaths and extensive economic damage. Recently, PTSD-related PIU has attracted widespread attention. Although both PTSD and PIU are prevalent adverse responses, the correlation between the two factors is unclear, especially in the context of Chinese society and social media. Some evidence suggests that individuals who are experiencing fear and seeking a safer environment may engage in avoidance behavior [6, 7]. In this case, the internet may serve as a safer environment because of its virtual nature and absence of visual cues, which may permit individuals to circumvent the actual context [6]. Thus, it is reasonable that PIU may result from fear regarding potential threats, and an increase in fear may be associated with an increase in PIU.

However, some studies refrained from conceptualizing PIU as a disease model and proposed that it is not a distinct psychopathological disorder, instead acting as a symptom of a preexisting mental disorder [7, 8, 9, 10]. Specifically, they hypothesized that greater fear of missing out (FOMO) may increase PIU and time spent online [8]. FOMO is characterized as a widespread anxiety that others may be engaging in gratifying experiences from which one is excluded, particularly on social networking platforms [9]. FOMO was often identified as a mediator connecting deficiencies in psychological needs to social media engagement. Research found that individuals with PTSD are more likely to perceive their social status as precarious and may seek validation from others, but these needs are hardly addressed, and social networking sites may lead to jealousy and diminished life satisfaction [10]. As a result, a potentially risky pattern may emerge when they are compelled to engage in passive social behavior, as evidenced by their PIU in social media, because of FOMO, which is initiated by depression. The association with depression may be further exacerbated by this sensation of social isolation [7]. Additionally, subsequent studies provided additional evidence that individuals with elevated FOMO scores often experience reduced psychological well-being [8, 11], and a correlation between PTSD and a cognitive process known as rumination has been identified [12, 13].

Rumination is a complicated process involving diverse elements, including both behavioral and cognitive dimensions. It mainly emphasizes the concept of adverse mental conditions and their ensuing consequences. This phenomenon has been recognized as a psychological vulnerability factor correlated with the development of depression and anxiety [14]. Rumination may alter the potential effect of trauma experiences on PTSD; however, there is a lack of discussion about the circumstances that could potentially affect this relationship [15]. A study revealed that individuals experiencing depression often engage in unproductive rumination, which tends to intensify with increased access to online information, indicating that rumination may serve as a significant response to an unforeseen stressful event [16]. Although rumination serves as a cognitive maintenance factor in PTSD, the predominant focus of current research remains on general forms of rumination such as negative abstract self-evaluations, rather than on rumination specifically related to post-disaster trauma. When examining internet usage and online information access in these populations, it remains ambiguous why trauma survivors engage in rumination despite its detrimental psychological effects. Overall, natural disasters are common in China every year, causing many negative mental effects on people. Our study aimed to clarify how Wenchuan earthquake-related experiences may lead to PIU, particularly examining the potential negative or positive effects of rumination and FOMO.

Given the background mentioned, we proposed the following hypotheses:

H1a: PTSD caused by the Wenchuan earthquake has a positive relationship with FOMO.

H1b: PTSD caused by the Wenchuan earthquake has a positive relationship with rumination.

H1c: PTSD caused by the Wenchuan earthquake has a positive relationship with PIU.

H2a: FOMO has a positive relationship with rumination.

H2b: FOMO has a positive relationship with PIU.

H2c: FOMO is indirectly and positively associated with PIU via rumination.

H2d: PTSD caused by the Wenchuan earthquake is indirectly and positively associated with PIU via FOMO.

H3a: Rumination has a positive relationship with PIU.

H3b: PTSD caused by the Wenchuan earthquake is indirectly and positively associated with PIU via rumination.

Combined with the above hypotheses, we reasonably put forward hypothesis H4:

H4: PTSD caused by the Wenchuan earthquake is indirectly and positively associated with PIU through a chain mediation model, with FOMO and rumination being the mediating variables.

**Fig. 1.**
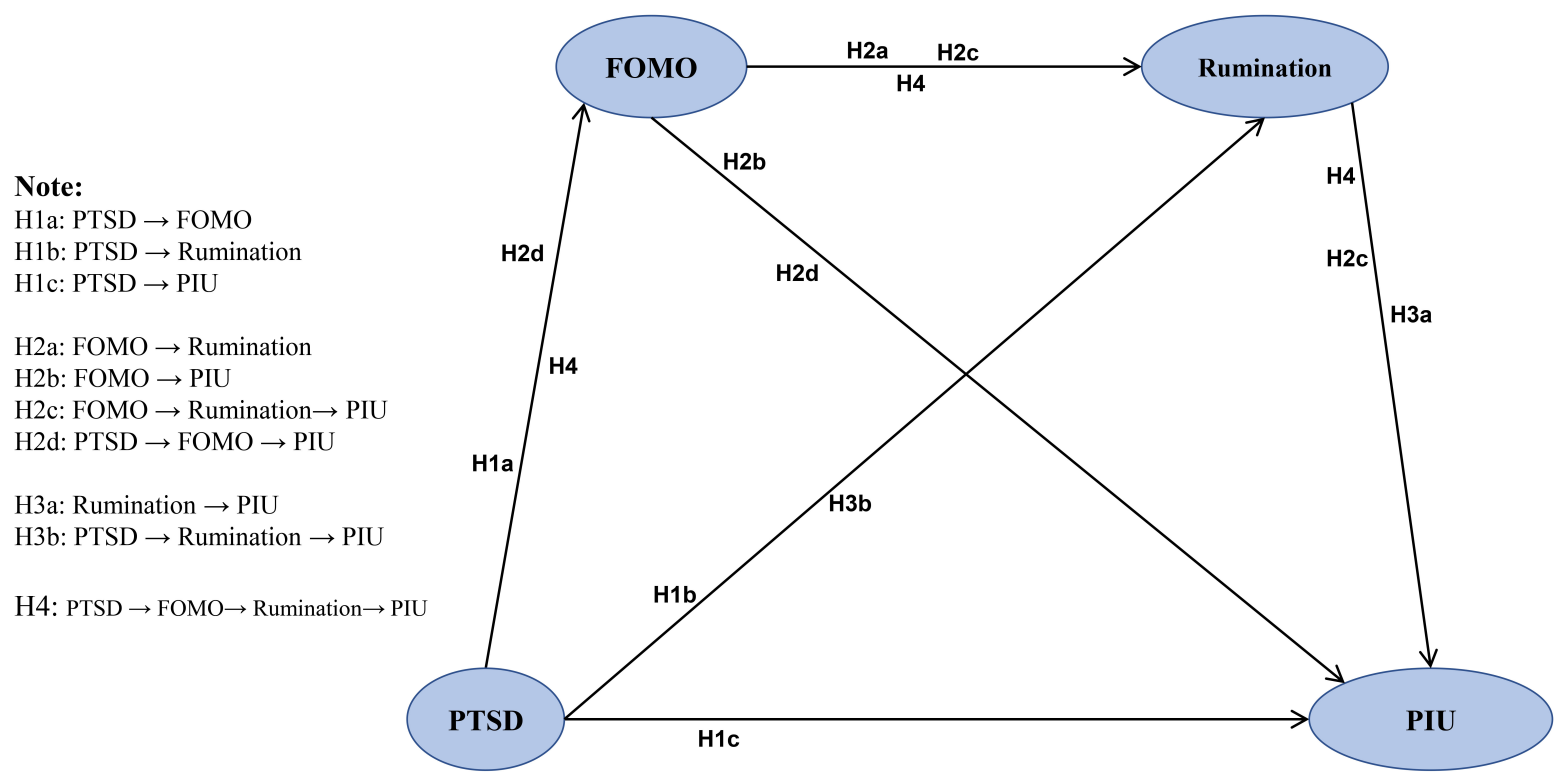
**Assumptive model**. PTSD, post-traumatic stress disorder; FOMO, fear of missing out; PIU, Problematic internet use.

A conceptual framework was formulated based on the above hypotheses, which is illustrated in Fig. 1.

Based on the above hypotheses and conceptual framework, we examined how the online communication environment has evolved in recent years among this specific population, and the challenges posed to internet social media managers. This study has several practical significances. The first was to examine the rise in general internet usage and particular online behaviors among survivors due to the prolonged stress caused by earthquakes. The second was to ascertain the differences between experienced and perceived mental stress among survivors regarding PIU and specific internet-related behaviors. Last but importantly, we applied a structural equation model to test the hypothesized model, examining the association between specific mental stress symptoms (PTSD, rumination), negative social media behavior (FOMO), and the increase in PIU in this population. Overall, the Wenchuan earthquake provided a unique opportunity to examine PIU in survivors in the context of catastrophe crisis communication and Chinese social media.

## 2. Methods

### 2.1 Participants

In the fall of 2023, we recruited research participants from Beichuan County, Sichuan, China, one of the most devastated regions of the Wenchuan earthquake. The Institutional Review Board at the authors’ institution approved the research during the data collection period. For the sampling method, convenience sampling was used. This method involved selecting the most readily available and willing students to engage in the study, thereby increasing the probability of accurate research completion. This is also aligned with the sampling methods of some other recent earthquake-PTSD studies [17, 18], which enabled us to enroll participants from the highest-density census via a cost-effective approach. We mailed and distributed 820 questionnaires; 665 responses were valid after excluding those with large amounts of missing responses or for which the participants did not meet demographic or inclusion criteria. For the inclusion and exclusion criteria, all participants had normal auditory and visual capabilities and had not consumed psychostimulants or any other substances influencing their cognitive performance. In addition, participants with other mental diseases related to PIU, such as obsessive-compulsive disorder, were excluded. Specifically, there were 218 men and 447 women between the ages of 25 and 60 years (M (Mean) = 38.98, SD (standard deviation) = 4.83) included in this survey.

### 2.2 Data Analysis

The structural relationship between variables was measured using the Partial Least Squares-Structural Equation Modelling (PLS-SEM) [19] method. Specifically, we performed descriptive statistical analysis and structural equation modelling using SmartPLS 4 (SmartPLS GmbH, Hamburg, Germany). No common method bias was found before the analysis process. Participants’ demographic characteristics were set as covariates to avoid potential disruptions caused by age, etc., and to obtain more accurate estimates of the relationships among the four psychological variables. For the measurement model (outer model), Cronbach’s α was employed to evaluate the internal consistency of scale-type measurement instruments. Satisfactory internal consistency is considered when Cronbach’s α is greater than 0.7 [20]. Additionally, composite reliability (CR) and average variance extracted (AVE) were used to examined convergent validity. We used the heterotrait-monotrait correlation ratio (HTMT) and the Fornell-Larcker criterion to conduct discriminant validity. According to Hair *et al*. [19], discriminant validity is proven when the value is below 0.85.

For analysis of the structural model (inner model), collinearity was assessed and the variance inflation factor (VIF) was considered to have an acceptable level when it was less than 5. Next, we examined the correlation and significance of structural model relationships by analyzing the path coefficients, t value and R-Squared (R^2^). To examine the significance of the hypotheses, we employed 5000 bootstraps at a 5% significance level (two-tailed).

## 3. Measures

### 3.1 PTSD Scale

We consulted previous peer-reviewed studies that utilized appropriate measures that were translated for Chinese participants. A frequently utilized measure is the adult version of the PTSD Checklist - Civilian Version [4], along with its subsequent modified versions [21], which have demonstrated strong validity and reliability in Chinese samples. This PTSD scale comprises 17 items, each using a five-point Likert scale from “1 = strongly disagree” to “5 = strongly agree”. An example item is “It is exceedingly distressing to contemplate”. Cronbach’s α was 0.91, which is in line with Tavakol and Dennick’s [22] interpretation of α and other study using the PTSD Checklist (PCL-C) scale [10]. The PTSD scale therefore has an excellent internal consistency in our study.

### 3.2 FOMO Scale

FOMO was evaluated via the Fear of Missing Out Scale, developed by Przybylski *et al*. [9]. This scale consists of 10 items and an example item is “I get worried when I find out my friends are having fun without me”. Higher scores indicate higher levels of FOMO. Cronbach’s α was 0.93, which is consistent with previous FOMO research in a Chinese context [10, 23], suggesting a good internal consistency.

### 3.3 Rumination Scale

The evaluation of rumination was performed using the Cognitive Emotion Regulation Questionnaire, specifically employing the Chinese version developed by Zhou and Wu [12]. The instrument is a 22-item questionnaire to evaluate various cognitive coping methods. All items are evaluated using a 5-point Likert scale, with “0 = completely disagree” and “4 = completely agree”. Higher scores indicate higher levels of rumination. Cronbach’s α was 0.86, suggesting an acceptable internal consistency in this study [22, 24].

### 3.4 Problematic Internet Use Scale

In this study, we adopted the Generalized Problematic Internet Use Scale 2 (GPIUS2) developed by Caplan [6]. The GPIUS2 comprises two novel factors: desire for online social interaction and inadequate self-regulation. This scale has 15 items and uses an eight-point Likert scale ranging from “1 = definitely disagree” to “8 = definitely agree”. An example item is “I have difficulty controlling the amount of time I spend online”. Higher scores indicate more severe levels of PIU. Cronbach’s α was 0.89, suggesting a good internal consistency [22, 24].

## 4. Results

Demographic variables included age, gender, education, and salary. As shown in Table 1, 665 participants were included in our study. It is important to note that the mean age was relatively high (M = 38.98, SD = 4.83) due to the inclusion of individuals who had independent thought at the time of the Wenchuan earthquake, which occurred 15 years ago. Meanwhile, the survey location was situated in the hilly region of southwestern China, where the economy is underdeveloped, and the per capita levels of education and income are quite low. The demographic results were also in line with previous Wenchuan earthquake studies [5, 10].

**Table 1.** **Statistical description of demographic characteristics (N = 665)**.

Demographic Characteristic		Number	Percentage
Age (years)			
	25–30	78	0.117
	31–40	224	0.337
	41–50	247	0.371
	>50	116	0.174
Gender			
	Male	218	0.328
	Female	447	0.672
Education			
	Junior high school (or below)	128	0.192
	Senior high school	212	0.318
	Undergraduate	138	0.208
	Postgraduate (Master’s and PhD)	187	0.281
Salary (CNY)			
	<4000	338	0.508
	4000–8000	307	0.462
	8000–12,000	18	0.027
	>12,000	2	0.003

1 CNY = 0.14 USD.

The evaluation of the measurement model was conducted by reliability and validity tests. Details of the construct reliability, factor loadings, composite reliability, and AVE can be found in the supplemental material. Cronbach’s α and factor loadings were above 0.7, indicating good consistency and validity in our study. The HTMT and Fornell-Larcker results are shown in Table 2. All values are less than 0.9. Overall, all the measures in this model demonstrated great psychometric properties.

**Table 2.** **Evaluation of the outer measurement model**.

		PTSD	FOMO	Rumination	PIU
Heterotrait-monotrait (HTMT) results					
	PTSD				
	FOMO	0.688			
	Rumination	0.252	0.321		
	PIU	0.587	0.448	0.545	
Fornell-Larcker results					
	PTSD	0.760			
	FOMO	0.728	0.691		
	Rumination	0.652	0.597	0.841	
	PIU	0.625	0.675	0.716	0.747

PTSD, post-traumatic stress disorder; FOMO, fear of missing out; PIU, problematic internet use.

Upon the estimated model meeting the outer model criteria, the structural model was evaluated, and R^2^ for the variables was analyzed. As shown in the supplemental material, R^2^ for FOMO was 0.446, R^2^ for rumination was 0.535, and R^2^ for PIU was 0.705. The coefficient of determination (R^2^) indicated that 71% of the variables affecting PIU can be explained by our model. PTSD could explain 45% of the variance in FOMO, while PTSD and FOMO could explain 54% (R^2^) of the variance in rumination.

The SmartPLS results for the direct effect of test outcomes for each variable regarding the path coefficient are shown in Table 3. We found that PTSD positively influenced FOMO (β = 0.315, *p* < 0.001), rumination (β = 0.279, *p* = 0.001), and PIU (β = 0.213, *p* < 0.001). FOMO also had positively relationships with rumination (β = 0.118, *p* = 0.017) and PIU (β = 0.585, *p* < 0.001). More details are shown in Table 3.

**Table 3.** **Path coefficients**.

	β	Se	t value	*p* value
PTSD → PIU	0.213	0.088	6.582	<0.001^*⁣**^
PTSD → FOMO	0.315	0.037	9.121	<0.001^*⁣**^
PTSD → Rumination	0.279	0.086	7.474	0.001^**^
FOMO → Rumination	0.118	0.079	7.389	0.017^*^
FOMO → PIU	0.585	0.09	9.656	<0.001^*⁣**^
Rumination → PIU	0.367	0.034	10.129	<0.001^*⁣**^

Note: ^**^p < 0.01, ^*⁣**^p < 0.001. The arrow means the referential relationship. For example, PTSD → PIU means PTSD has a significant effect on PIU.

For the mediation test, 5000 bootstrap simulation analyses were conducted. This study was performed to ascertain percentile confidence ranges for the bias correction of indirect effects. The mediation test results are shown in Table 4. PTSD can both directly (β = 0.279, *p* = 0.001) and indirectly (through FOMO, with β = 0.194, *p* = 0.001) influence rumination. The results also indicate that PTSD can have a significant influence on PIU. Concerning the indirect effect, PTSD had positive relationships with PIU via both FOMO (β = 0.08, 95% CI [0.037, 0.144], *p* = 0.005) and rumination (β = 0.093, 95% CI [0.032, 0.139], *p* = 0.002). Meanwhile, PTSD had a positively significant effect on PIU by mediating FOMO and rumination to form a chain mediation model (β = 0.081, 95% CI [0.010, 0.039], *p* = 0.002). Overall, for the relationship between PTSD and PIU, direct (β = 0.213, 95% CI [0.025, 0.245], *p* < 0.001) and indirect effects (through FOMO, with β = 0.08, 95% CI [0.037, 0.144], *p* = 0.005; through rumination, with β = 0.093, 95% CI [0.032, 0.139], *p* = 0.002) comprised 45.6% and 54.4% of the total effect, respectively. Additionally, FOMO was also found to have both direct (β = 0.585, *p* < 0.001) and indirect (through rumination, with β = 0.086, *p* < 0.001) effects on PIU, and they constituted 87.2% and 12.8% of the overall effect.

**Table 4.** **Mediation analysis results**.

Mediation Analysis		β	Se	t	*p*	95% CI	
						Lower	Upper
Indirect effect							
	PTSD → FOMO → Rumination	0.194	0.039	3.443	<0.001^*⁣**^	0.041	0.152
	PTSD → FOMO → Rumination→ PIU	0.081	0.009	3.029	0.002^**^	0.010	0.039
	PTSD → FOMO → PIU	0.080	0.035	3.774	0.005^**^	0.037	0.144
	PTSD → Rumination → PIU	0.093	0.024	3.935	0.002^**^	0.032	0.139
	FOMO → Rumination → PIU	0.086	0.019	4.615	<0.001^*⁣**^	0.054	0.129
Direct effect							
	FOMO → PIU	0.585	0.09	9.656	<0.001^*⁣**^	0.019	0.238
	PTSD → PIU	0.213	0.088	6.582	<0.001^*⁣**^	0.025	0.245
Total effect							
	FOMO → PIU	0.671	0.059	11.121	<0.001^*⁣**^	0.307	0.591
	PTSD → PIU	0.467	0.063	6.954	<0.001^*⁣**^	0.227	0.443

Note: ^**^p < 0.01, ^*⁣**^p < 0.001. The arrow means the referential relationship.

## 5. Discussion

In this study, we found that PTSD, FOMO, and rumination were significantly and positively related to PIU, supporting H1c, H2b, H3a, and H3b. The results also demonstrated that these psychological factors may be risk factors for PIU. Additionally, PTSD is positively associated with FOMO and rumination respectively, supporting H1a and H1b. To our knowledge, this study is the first to examine the mediating role of FOMO and rumination in the relationship between PIU and PTSD, among adults after the Wenchuan earthquake in China.

Our findings revealed that Wenchuan earthquake-related PTSD experiences may directly lead to PIU, which is consistent with several analogous studies concerning the media psychological consequences of disasters [25, 26, 27]. Specifically, one study [26] regarding post-earthquake analysis in Irpinia, Italy, found a long adaptation process on the human and media environment begins after each disaster. Research by Vukovic *et al*. [27] among Bosnian refugees also found that PTSD and chronic illness are prevalent in the refugee experience and can adversely affect the level of social media usage. Our findings also support the theory put forward by Janoff-Bulman [28], which proposed that PTSD can challenge people’s stable belief systems such that more negative cognition and emotion emerges, and in turn more PIU symptoms are elicited. Moreover, Janoff-Bulman [28] underscored the significant impact of negative cognitive processes, such as rumination, on the development of PTSD. It is hypothesized that rumination may contribute to PIU by the following mechanisms. First, the impact of negative mood on cognition can be exacerbated by rumination, which is why traumatized individuals are more likely to employ negative memories and thoughts that are influenced by negative mood to comprehend their current circumstances. Second, rumination may hinder the process of effective problem solving, and people may think more pessimistically and uncontrollably when using the internet.

However, the mediation effect size of rumination was comparatively modest. A possible reason may be the minimal effect size of earthquake-related experiences on rumination. As this earthquake occurred more than 10 years ago, actions such as psychological interventions and social support at subsequent times may have fostered resilience [16], which could mitigate the tendency to ruminate.

The most delightful result is the findings of the chain mediation model between PTSD, FOMO, rumination, and PIU (hypothesis H4). Social internet networks during disasters are crucial for maintaining interpersonal relationships, facilitating communication, and fulfilling educational and professional responsibilities. However, excessive usage of social networks leads to unwanted repercussions, such as increasing online activity and the rise of problematic social media use. In our study, FOMO and rumination were also increased in the PTSD population. Uncontrolled online behaviors can turn into compulsive usage during prolonged stress, which may be associated with the regulation of unpleasant emotional states. Overall, the 2008 Wenchuan earthquake offers a distinctive chance to analyze how people have been using the internet as a medium for crisis communication since it happened. Despite nearly a decade passing since the Wenchuan earthquake, it is pertinent to examine the actions of internet users for several reasons. In our study, the information processing behavior of earthquake survivors has been extensively examined in the context of disaster warnings or experimental settings. Additionally, investigating their communication behavior from prior to the era of social media can facilitate the identification of persistent characteristics in communication practices when contrasted with current circumstances. Last but not least, in China, the Wenchuan earthquake is almost unique in its extensive societal impact, unparalleled by other disasters in the digital age [5]. Some factors that may mediate the relationship among mental health and internet usage, as well as online activities during stressful times, warrant further investigation and consideration when implementing targeted interventions to alleviate psychological distress and assist at-risk populations. Our findings demonstrated the influence of PTSD on PIU and FOMO and rumination as reactions to extended stressful experiences in Wenchuan earthquake survivors.

## 6. Limitations and Implications

While the findings presented herein support the proposed models, some methodological limitations should be addressed that may influence conclusions based on the study. First, researching problematic behavior presents logistical and ethical challenges, particularly in obtaining objective metrics, as stated by previous related studies. Each measure depended on self-reporting instead of diagnostic interviews or other reports. These may be influenced by the mental states of participants, perhaps resulting in reporting bias. In addition, this study’s results relied on the convenience sampling and mailing method. While consistent with the research methodology of numerous post-disaster media psychology studies [17, 18, 26, 27], the conclusions warrant cautious interpretation. However, overall, the importance of studying the survivor’s community in Beichuan, representing the most severely affected region of the Wenchuan earthquake, is apparent. Second, the evaluation of PIU was based on limited items compared with some comprehensive PIU research [29, 30], indicating that this measure may incompletely reflect every problematic situation in the social media context. Third, considering a lack of baseline assessments, the dynamic change in PIU may not be solely attributed to the impact of the stressful event (e.g., the earthquake). Fourth, some psychopathological problems (e.g., depression and anxiety) [2] may also be associated with PIU, but the relationship between these mental states and trajectories of PIU were not examined in this study. Last, it is worth addressing that this study is cross-sectional rather than longitudinal, which is less likely to formally establish causality. However, the SEM method indeed enables us to determine whether our data support the causal associations that have been hypothesized. The hypothesized model was substantially supported by the SEM results in the present research.

Despite the above limitations, our results incorporate significant theoretical and practical implications. This study may be the first to examine correlations among PTSD, FOMO, rumination, and PIU, thus making a substantial contribution to the academic literature. This research and its conclusions are important due to the dramatic increase in the occurrence of problematic web use in the social media era.

Regarding practical implications, the findings may provide important information for policymakers and local government. We found that many participants showed serious PIU even many years after the earthquake. A plausible reason is that the Wenchuan earthquake, measuring 8.0 on the Richter scale, inflicted significant damage to the physical infrastructure in the affected regions (like Beichuan county) [10]. At that time, people had restricted access to the internet. As post-disaster rehabilitation progresses, the physical infrastructure in earthquake-affected areas is being gradually rebuilt, thereby increasing people’s access to the internet. For local government, we highly propose the implementation of campaigns to raise awareness of internet use and help people promote media literacy. It is also recommended to conduct educational and intervention policies to improve disaster preparedness beliefs. Nursing interventions that consider individuals’ personal characteristics and spiritual well-being are encouraged. Additionally, media workers should adopt a more aggressive stance in promoting awareness regarding PIU and its ramifications. To alleviate the negative effects of PIU on individuals with PTSD, subsequent research should investigate relevant psychological interventions, such as positive thinking therapy [31]. Academic researchers should further investigate the correlations between individual differences, such as personalities, and specific platform characteristics to enhance understanding of who is most susceptible to PIU. The above implications are quite instructive for accelerating the restoration to both internet use and mental health normalcy for individuals suffering from earthquakes.

## 7. Conclusions

This study investigated online usage and media psychology among the survivors of a catastrophic earthquake in China. We found that PIU has become one of the most serious symptoms among those participants. PTSD has a positive relationship with PIU, via both direct and indirect pathways. FOMO and rumination may act as a significant mediating role in the model, and they also have a positive association with PIU. This study provides insights into the PTSD population’s problematic online behavior and negative mental state in the social media era. Future intervention and academic research could be further undertaken based on the findings and implications of this study.

## Availability of Data and Materials

The data that support the findings are available upon reasonable request from the corresponding author.

## References

[1] Zhou X, Zhen R, Wu X (2018). Trajectories of Problematic Internet Use among adolescents over time since Wenchuan earthquake. *Computers in Human Behavior*.

[2] Kovačić Petrović Z, Peraica T, Blažev M, Tomašić L, Kozarić-Kovačić D (2022). Problematic Internet Use, Anxiety, Depression, and Stress Symptoms in Adults with COVID-19 Pandemic and Earthquake Experience: Insights from Croatian Online Survey. *Cyberpsychology, Behavior and Social Networking*.

[3] Kovačić Petrović Z, Peraica T, Blažev M, Kozarić-Kovačić D (2023). Association between problematic Internet use and specific Internet activities and COVID-19- and earthquake-related stress, anxiety, and depression symptoms among Croatian young adults. *Frontiers in Psychiatry*.

[4] Weathers FW, Litz B, Herman D, Juska J, Keane T (1994). PTSD Checklist-Civilian Version. *Journal of Occupational Health Psychology*.

[5] Fan X, Juang CH, Wasowski J, Huang R, Xu Q, Scaringi G (2018). What we have learned from the 2008 Wenchuan Earthquake and its aftermath: A decade of research and challenges. *Engineering Geology*.

[6] Caplan SE (2010). Theory and measurement of generalized problematic Internet use: A two-step approach. *Computers in Human Behavior*.

[7] Sela Y, Zach M, Amichay-Hamburger Y, Mishali M, Omer H (2020). Family environment and problematic internet use among adolescents: The mediating roles of depression and Fear of Missing Out. *Computers in Human Behavior*.

[8] Roberts JA, David ME (2020). The Social Media Party: Fear of Missing Out (FoMO), Social Media Intensity, Connection, and Well-Being. *International Journal of Human–Computer Interaction*.

[9] Przybylski AK, Murayama K, DeHaan CR, Gladwell V (2013). Motivational, emotional, and behavioral correlates of fear of missing out. *Computers in Human Behavior*.

[10] Gong C, Ren Y (2023). PTSD, FOMO and fake news beliefs: a cross-sectional study of Wenchuan earthquake survivors. *BMC Public Health*.

[11] Hattingh M, Dhir A, Ractham P, Ferraris A, Yahiaoui D (2022). Factors mediating social media-induced fear of missing out (FoMO) and social media fatigue: A comparative study among Instagram and Snapchat users. *Technological Forecasting and Social Change*.

[12] Zhou X, Wu X (2016). The relationship between rumination, posttraumatic stress disorder, and posttraumatic growth among Chinese adolescents after earthquake: A longitudinal study. *Journal of Affective Disorders*.

[13] Bernstein EE, Heeren A, McNally RJ (2019). Reexamining trait rumination as a system of repetitive negative thoughts: A network analysis. *Journal of Behavior Therapy and Experimental Psychiatry*.

[14] Segerstrom SC, Tsao JC, Alden LE, Craske MG (2000). Worry and rumination: Repetitive thought as a concomitant and predictor of negative mood. *Cognitive therapy and Research*.

[15] Carpenter KM, Fowler JM, Maxwell GL, Andersen BL (2010). Direct and buffering effects of social support among gynecologic cancer survivors. *Annals of Behavioral Medicine: a Publication of the Society of Behavioral Medicine*.

[16] Astuti SW (2022). Social media effect on loneliness, rumination and social comparison. *Webology*.

[17] Messiah A, Acuna JM, Castro G, de la Vega PR, Vaiva G, Shultz J (2014). Mental health impact of the 2010 Haiti earthquake on the Miami Haitian population: A random-sample survey. *Disaster Health*.

[18] Kaya M, Bayram SS (2024). Determining the impact of earthquakes on university students’ hope and anxiety levels. *International Journal of Disaster Risk Reduction*.

[19] Hair JF, Ringle CM, Sarstedt M (2011). PLS-SEM: Indeed a Silver Bullet. *Journal of Marketing Theory and Practice*.

[20] Bland JM, Altman DG (1997). Cronbach’s alpha. *BMJ (Clinical Research Ed.)*.

[21] Xie X, Wang Y, Wang P, Zhao F, Lei L (2018). Basic psychological needs satisfaction and fear of missing out: Friend support moderated the mediating effect of individual relative deprivation. *Psychiatry Research*.

[22] Tavakol M, Dennick R (2011). Making sense of Cronbach’s alpha. *International Journal of Medical Education*.

[23] Li L, Niu Z, Mei S, Griffiths MD (2022). A network analysis approach to the relationship between fear of missing out (FoMO), smartphone addiction, and social networking site use among a sample of Chinese university students. *Computers in Human Behavior*.

[24] Cronbach LJ (1951). Coefficient alpha and the internal structure of tests. *Psychometrika*.

[25] Woolverton GA, Stevens C, Hahm HC, Liu CH (2024). Rates and psychological stress predictors of problematic internet use (PIU) during the COVID-19 pandemic in a racially diverse sample of young adults. *Anxiety, Stress, and Coping*.

[26] Moscaritolo GI, Forino G (2024). Adaptive Disaster Memories: Voices from the Post-earthquake Irpinia (23 November 1980). *Disasters and Changes in Society and Politics*.

[27] Vukovic IS, Valeriani G, Britvic D, Jovanovic N, Mollica R (2024). Marital loss, gender and their association with mental health and physical health outcomes in Bosnian refugees: lesson reminder in a time of war. *Rivista Di Psichiatria*.

[28] Janoff-Bulman R (2010). Shattered Assumptions.

[29] Chen L, Yang Y, Su W, Zheng L, Ding C, Potenza MN (2018). The relationship between sexual sensation seeking and problematic Internet pornography use: A moderated mediation model examining roles of online sexual activities and the third-person effect. *Journal of Behavioral Addictions*.

[30] Cheng X, Liu J, Li J, Hu Z (2023). COVID-19 lockdown stress and problematic social networking sites use among quarantined college students in China: A chain mediation model based on the stressor-strain-outcome framework. *Addictive Behaviors*.

[31] Abrahams HJG, Gielissen MFM, Donders RRT, Goedendorp MM, van der Wouw AJ, Verhagen CAHHVM (2017). The efficacy of Internet-based cognitive behavioral therapy for severely fatigued survivors of breast cancer compared with care as usual: A randomized controlled trial. *Cancer*.

